# Identification of Candidate Variants Associated With Bone Weight Using Whole Genome Sequence in Beef Cattle

**DOI:** 10.3389/fgene.2021.750746

**Published:** 2021-11-29

**Authors:** Qunhao Niu, Tianliu Zhang, Ling Xu, Tianzhen Wang, Zezhao Wang, Bo Zhu, Xue Gao, Yan Chen, Lupei Zhang, Huijiang Gao, Junya Li, Lingyang Xu

**Affiliations:** Key Laboratory of Animal Genetics Breeding and Reproduction, Ministry of Agriculture and Rural Affairs, Institute of Animal Sciences, Chinese Academy of Agricultural Sciences, Beijing, China

**Keywords:** genetic architecture, imputed sequence variants, bone weight, Bayesian fine-mapping, beef cattle

## Abstract

Bone weight is critical to affect body conformation and stature in cattle. In this study, we conducted a genome-wide association study for bone weight in Chinese Simmental beef cattle based on the imputed sequence variants. We identified 364 variants associated with bone weight, while 350 of them were not included in the Illumina BovineHD SNP array, and several candidate genes and GO terms were captured to be associated with bone weight. Remarkably, we identified four potential variants in a candidate region on BTA6 using Bayesian fine-mapping. Several important candidate genes were captured, including *LAP3*, *MED28*, *NCAPG*, *LCORL*, *SLIT2*, and *IBSP*, which have been previously reported to be associated with carcass traits, body measurements, and growth traits. Notably, we found that the transcription factors related to *MED28* and *LCORL* showed high conservation across multiple species. Our findings provide some valuable information for understanding the genetic basis of body stature in beef cattle.

## Introduction

Cattle, as one of the most important farm animals, provide numerous meat products for high-quality protein. Carcass merit traits are a key factor that directly affects meat yield, and their performance is highly connected with stature size (Sieber et al., 1988; Albertí et al., 2008; Ozkaya and Bozkurt 2008). Bone weight can reflect the size of stature and the skeleton frame (Berg and Butterfield 1966; Chumlea et al., 2002; Conroy et al., 2010) and is involved in the respiratory disease and feed efficiency in cattle (Snowder et al., 2007; Mader et al., 2009). Meanwhile, many economically important traits (carcass weight, meat yield, etc.) were significantly associated with bone weight (Pabiou et al., 2012). Therefore, elucidation of the genetic basis for bone weight can provide valuable information into understanding the bone development (Browning and Browning 2009), as well as exploring the potential the molecular mechanism of stature size in cattle.

Over the past few years, genome-wide association studies (GWAS) have been widely utilized to identify quantitative trait loci (QTLs) associated with complex traits in cattle (Wu et al., 2013; Bouwman et al., 2018; Fang and Pausch 2019). Several QTLs relevant to stature, body size, growth, and carcass traits have been revealed in many previous studies, including *PLAG1* (stature) (Karim et al., 2011; Bouwman et al., 2018) and *NCAPG-LCORL* (body size, carcass weight and feed efficiency) (Lindholm-Perry et al., 2011; Zhang et al., 2016; Chen et al., 2020).

Whole genome sequencing (WGS) theoretically contains all the causative mutations underlying complex traits; thus, this technology can avoid the limitation of SNP array designed by pre-selected variants and boost the ability to identify the novel candidate variants (Daetwyler et al., 2014). Recently, it is feasible and cost-effective to impute the low-density arrays to sequence level by using the small sequenced population as the reference. Imputation approach has been widely applied to detect the candidate variants for important traits in cattle (Bordbar et al., 2019; Purfield et al., 2019; Zhang et al., 2020). However, GWAS using sequencing variants may result in plenty of variants concentrating in a genomic region because of the high level of linkage disequilibrium (LD) among identified regions, which makes it difficult to accurately locate GWAS signals. Therefore, many approaches were proposed to identify the truly candidate variant from hundreds of significant variants with the high levels of LD, including condition and joint analysis (Yang et al., 2012) and Bayesian fine-mapping (Jiang et al., 2019). These strategies have been applied to precisely identify the causal variants and capture the secondary association signals for economically important traits in cattle (Fang et al., 2019; Freebern et al., 2020; Sanchez et al., 2020; Warburton et al., 2020).

Bone weight is important to body conformation and stature. Many previous studies have explored the genetic basis of bone weight using Illumina BovineHD SNP BeadChip based on different models. Several candidate genes including *LCORL*, *NCAPG*, *LAP3*, *RIMS2*, and *SLIT2* were detected for bone weight (Xia et al., 2017; Chang et al., 2018; Miao et al., 2018; Chang et al., 2019; An et al., 2020). These genes have also been identified for some other traits relevant to stature size, such as live weight, carcass weight, and body measurements (Song et al., 2016; An et al., 2018; Zhang W. et al., 2018). Despite the fact that several candidate genes and variants for bone weight have been detected in previous studies, the understanding for genetic architecture of bone weight remains to be improved with the applications of whole genome sequencing technology and advanced analysis methods.

In the current study, to explore the quantitative trait nucleotides (QTN) associated with bone weight, we firstly conducted a GWAS based on imputed whole genome sequencing variants. Using fine-mapping analysis, we further located the candidate variant within GWAS signal in the candidate region based on the Bayesian fine-mapping analysis.

## Materials and Methods

### Animals and Phenotypes

A total of 1,233 Chinese Simmental beef cattle from Ulgai, Xilingol League were fed with the same conditions. A more detailed description of the feeding and management has been described previously (Zhu et al., 2017). The cattle were slaughtered at an average age of approximately 20 months. During slaughter, we measured the traits in strict accordance with the guidelines proposed by the Institutional Meat purchase Specifications for fresh beef (Zhu et al., 2017). Bone weight for each individual was the total weight of the bones in all the forequarter and hindquarter joints. The phenotypes of bone weight were adjusted using the general linear model. Farm, year, and sex were considered as the fixed effect, and weight before fattening and fattening days were considered as covariates. Then, we considered the residuals as the pre-adjusted phenotype for the subsequent analysis.

### Genotype and Imputed Sequence Variants

In total, 1,233 individuals were genotyped with the Illumina BovineHD 770 K SNP array. The SNP markers were pre-processed using PLINK v1.9 (Purcell et al., 2007; Chang et al., 2015). High-quality SNPs were selected based on the proportion of missing genotypes (<0.05), minor allele frequency (>0.01), and Hardy–Weinberg Equilibrium test (*p* > 10e-6). Also, individuals with missing genotypes (>10%) were excluded. After quality controls, a total of 641,277 autosomal SNPs were available for the subsequent imputation.

The BovineHD genotype of 1,233 individuals was imputed to whole genome sequence level based on the reference population from the unrelated 44 individuals. More detailed information about the selection of sequencing individuals and sequencing variants calling were provided in a previous study (Niu et al., 2021). We performed the imputation from SNP array to sequencing level using BEAGLE v4.1 (Browning and Browning 2007). After the imputation, a total number of 16,165,263 SNPs were obtained. The quality controls were performed including minor allele frequency (MAF) > 0.005 and imputation accuracy (*R* ^2^) > 0.1. Finally, 12,102,431 imputed sequencing SNPs were retained for the association analysis.

### Genome-Wide Association Analyses

The association study for bone weight was performed using the mixed linear model in GCTA v1.93.1 software (Yang et al., 2011; Yang et al., 2014). The model is:

$$y_{i}=\mu +b_{j}x_{ij}+g_{i}+e_{i}$$ (1)

where *y*_*i*_ is the pre-adjusted phenotypic value of the *i*th animal, *µ* is the mean, *b*_*j*_ is the allele substitution effect of the *j*th SNP, *x*_*ij*_ is the *j*th SNP genotype of animal *i* and *x*_*ij*_ is coded as 0, 1, and 2 for genotypes A_1_A_1_, A_1_A_2_, and A_2_A_2_; *g*_*i*_ is the additive polygenetic effect of the *i*th animal, which was assumed to be distributed as $g_{i}∼N\left(0,G\sigma_{a}^{2}\right)$ . *G* is the additive genetic relationship matrix that was constructed based on all imputed SNPs using the GREML option in GCTA (Yang et al., 2010), and $\sigma_{a}^{2}$ is the genetic variance explained by all SNPs. *e*_*i*_ is the random residual effect, and it was assumed to be distributed as $e_{i}∼\text{N}\left(\text{0,I}\sigma_{\text{e}}^{2}\right)$ , where I is the identity matrix and $\sigma_{e}^{2}$ is the residual variance. The heritability for bone weight was estimated using GCTA-GREML based on BovineHD SNP array and imputed sequencing variants. The genomic heritability was computed by $h^{2}=\frac{\sigma_{a}^{2}}{\sigma_{a}^{2}+\sigma_{e}^{2}}$ .

The percentage of phenotypic variance and genetic variance explained by each significant SNP were calculated by ${\text{Var}}_{\text{p}}\left(\%\right)=2pq\beta^{2}/\sigma_{p}^{2}*100\%$ and $Var_{g}\left(\%\right)=2pq\beta^{2}/\sigma_{a}^{2}*100\%$ , respectively, where *p* and *q* are the allele frequencies for each SNP; is the SNP allele substitution effect; $\sigma_{p}^{2}$ is the phenotypic variance; and $\sigma_{a}^{2}$ is the additive genetic variance. Here, we adopt the Bonferroni correction to set the adjusted threshold of genome-wide significant *p*-values (Hayes 2013). Thus, the genome-wide significance and suggestive significance threshold were set as 9.55e-09 (*p* = 0.01/N) and 9.55e-07(*p* = 1/N), respectively, where N denotes the effective number of independent variants (a total of 1,047,272) after removing SNPs based on linkage disequilibrium (LD).

### Comparison Analysis of Candidate Variants Between Previous and Current Studies

To explore evidence of the identified variants and genes from previous studies, we summarized the association results obtained by different methods based on Illumina BovineHD array including single marker association, multi-marker association, and gene-based association, and we compared their results with the finding in our study (Xia et al., 2017; Chang et al., 2018; Miao et al., 2018; Chang et al., 2019; An et al., 2020). The coordination of candidate SNPs or regions was determined according to the UMD 3.1 genome assembly.

### Bayesian Fine-Mapping Analyses

To capture the independent GWAS signals in the candidate region and distinguish the possible causal variant in each signal, we performed the Bayesian fine-mapping analysis using BFMAP software (Jiang et al., 2019). The posterior probability of causality (PPC) for each variant and *p*-value of causality for independent association signals within candidate QTL regions were calculated to evaluate the causality of these variants. The candidate variants were determined by the causality *p*-value and the posterior probability in each signal. More information about BFMAP can be found at https://github.com/jiang18/bfmap.

### Candidate Variants and Functional Enrichment Analyses

The candidate genes identified at suggestive threshold were located based on the UCSC database (http://genome.ucsc.edu/) in a 100-kb window (50 kb upstream and downstream of the variants). We obtained a list of candidate genes by integrating the results of current and previous studies. To explore the GO terms of candidate genes, gene set enrichment analysis was conducted using g:Profiler (Raudvere et al., 2019). The significance of GO terms was determined by the pre-adjusted g: SCS threshold (*p* < 0.05) in g: Profiler. The cattle QTL information was retrieved from database https://www.animalgenome.org/cgi-bin/QTLdb/BT/index (release 45, August 23, 2021). In addition, to assess the impact of SNPs on the regulation of gene expression, the information for the transcription factors (TFs) and the expression patterns of candidate genes were obtained from AnimalTFDB3.0 (http://bioinfo.life.hust.edu.cn/AnimalTFDB/) and Bgee database (https://bgee.org/).

## Results

### GWAS Analyses for Bone Weight Using Imputed Sequence Variants

**FIGURE 1:**
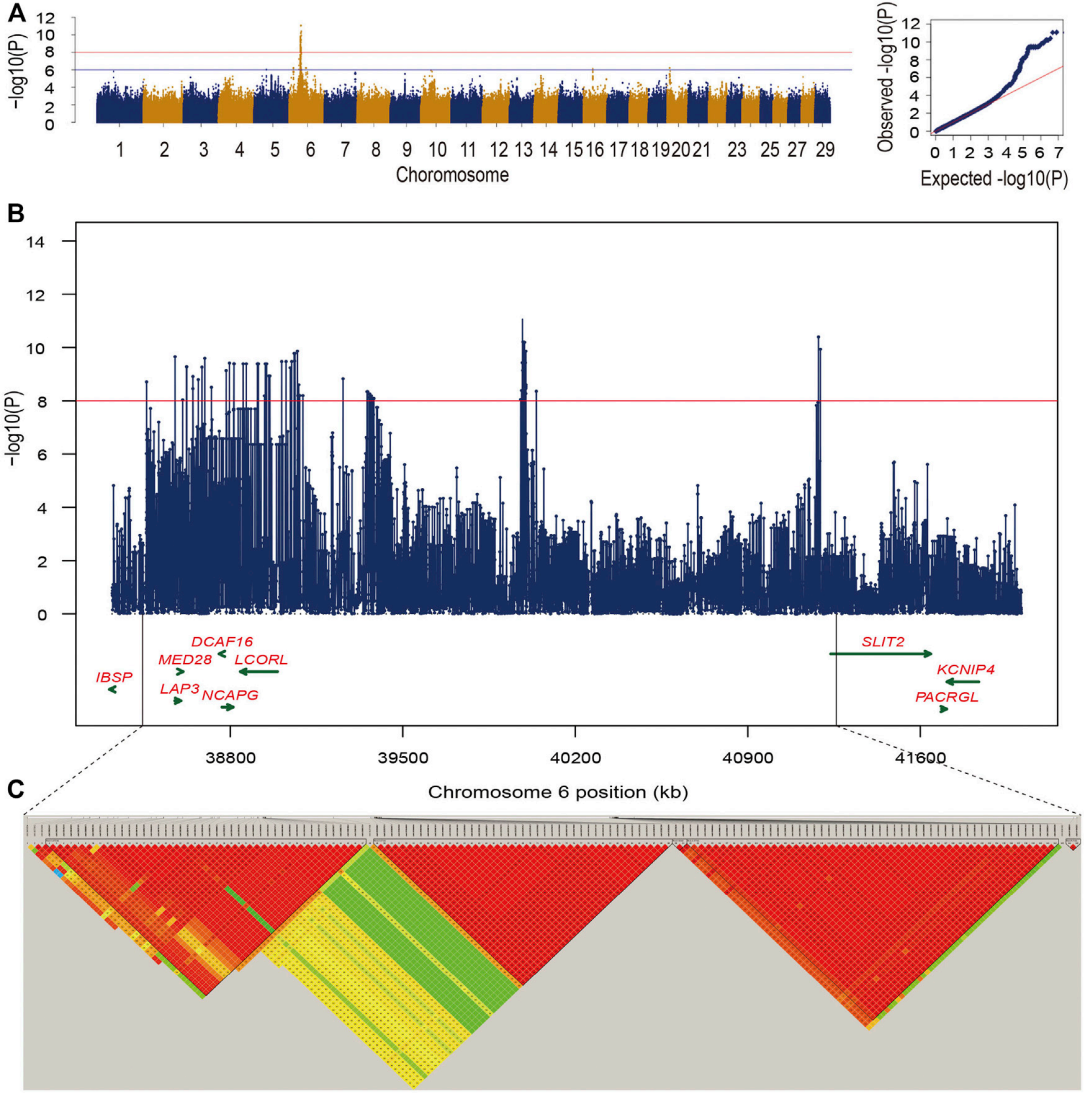
**(A)** Manhattan plot and Q-Q plot for −log_10_ (*p*-values) of SNPs from single-trait GWAS for bone weight. The two lines indicated significance threshold (*p* = 9.55e-09) and suggestive significance threshold (*p* = 9.55e-07), respectively. **(B)** The zoom-in plot of candidate regions at 38.3–42.0 Mb on BTA6. **(C)** Five high LD blocks at the candidate region on BTA6.

In this study, we found that the heritability for bone weight estimated by Illumina BovineHD SNP array and the imputed sequence variants were 0.43 ± 0.07 and 0.44 ± 0.08, respectively. Using association analysis based on the imputed sequence variants, we totally identified 145 candidate variants for bone weight under the significant threshold. Manhattan plot presents the GWAS signals across the genome for bone weight, and the QQ-plot shows that mixed linear model is suitable for the analysis of our data (Figure 1A). Under the suggestive level, we found that 364 candidate variants were distributed on four chromosomes including BTA5, BTA6, BTA16, and BTA20, while most of the candidate variants were detected on BTA6.

**FIGURE 2:**
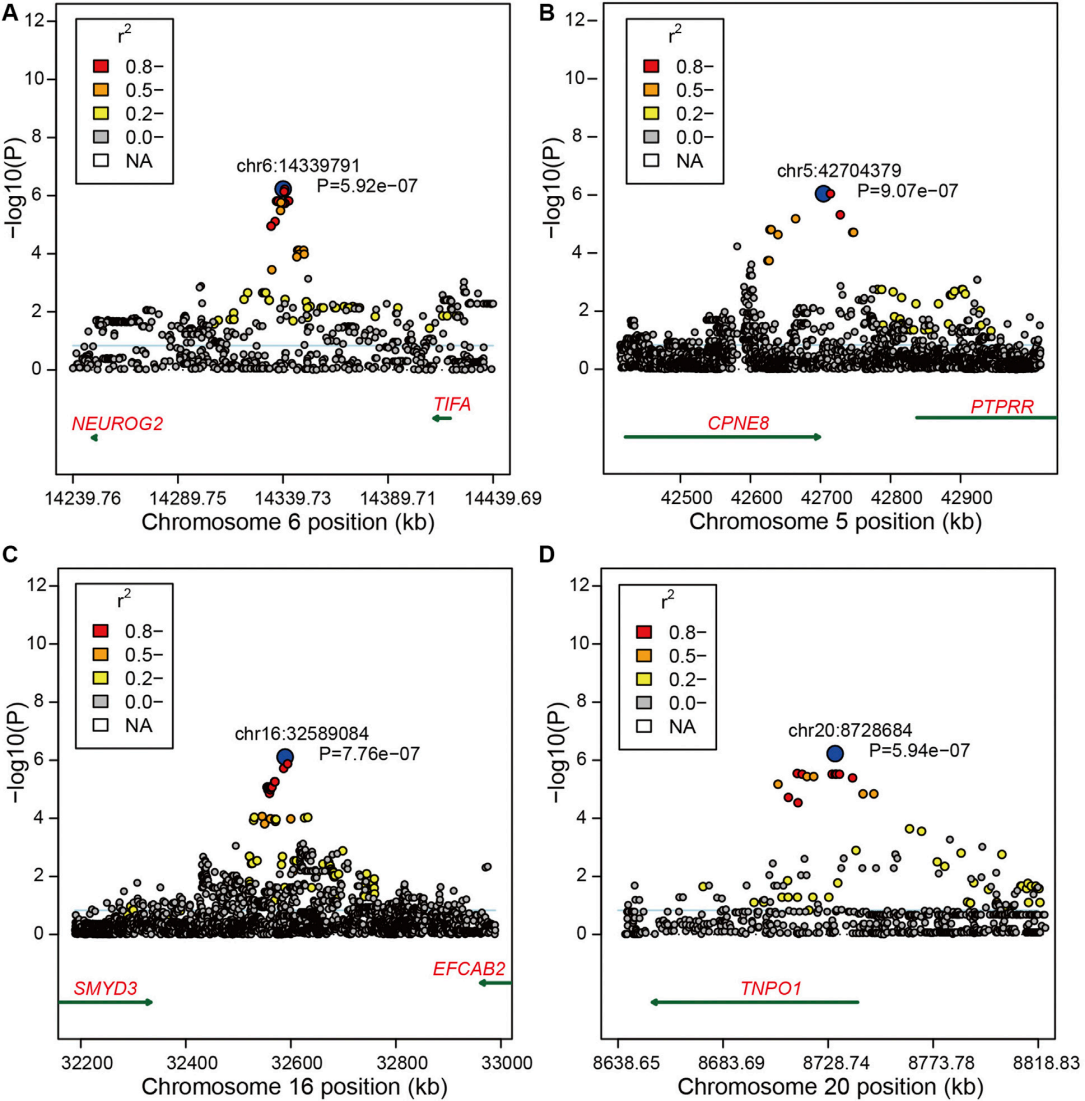
Region plots of candidate regions for bone weight. **(A)** Region plots of candidate regions at 14.23–14.44 Mb nearby SNP chr6:14339791 on BTA6. **(B)** Region plots of candidate regions at 42.50–42.90 Mb nearby SNP chr5:42704379 on BTA5. **(C)** Region plots of candidate regions at 32.20–33.00 Mb nearby SNP chr16:32589084 on BTA16. **(D)** Region plots of candidate regions at 8.64–8.81 Mb nearby SNP chr20:8728684 on BTA15. The top SNP was marked by blue circles. The extent of LD between the top SNP and the surrounding SNPs were presented in different colors. The candidate genes were shown under the *x*-axis = 0, and the dark green arrow represents the strand orientation.

Moreover, we found 145 significant variants located on BTA6 with the strong LD level, and five blocks with high LD were obtained in this region (Figures 1B,C). The most significant variant (chr6:39989730) contributing to ∼12.33% of the additive genetic variance and 5.46% of phenotypic variance is located at the intergenic region; however, no candidate gene was identified in the region at 50 kb upstream and downstream of this SNP. We also captured 10 significant intron variants within four candidate genes including *LAP3*, *MED28*, *NCAPG*, and *LCORL* (Supplementary Table S1). After zooming in the windows for 50 kb upstream and downstream, we observed an intergenic variant (chr6:38723514) with a MAF of 0.38, located at the 30,901 bp downstream of *DCAF16*, contributing to ∼9.35% of the genetic variance and 4.14% of phenotypic variance. Meanwhile, a variant (chr6:41186810) with the *p*-value of 4.02e-11 at the 49,460 bp downstream of *SLIT2* can explain ∼10.6% of total additive genetic variance (Supplementary Table S1). Under the suggestive threshold, we captured three genes containing *ABCG2*, *PKD2*, and *FAM13A* on BTA6 (Supplementary Table S2). An intergenic variant (*p*-value = 5.92e-07) was captured nearside two candidate genes including *NEUROG* and *TIFA*, while a weak LD level was observed around the 1-Mb regions (Figure 2A).

Based on the suggestive threshold, we identified two, one, and one candidate SNPs for bone weight on BTA5, BTA16, and BTA20 (Supplementary Table S2). Moreover, the suggestive variant (chr5:42704379) with a *p*-value of 9.07e-07 on BTA5 was located at 5,996 bp of *CPNE8*, showing a relatively strong LD with other SNPs close to *CPNE8* (Figure 2B). Another candidate variant on BTA16 was captured showing a strong LD with nearby SNPs, while no gene was identified nearby this region (Figure 2C). On BTA20, we identified a variant (chr20:8728684) within *TNPO1* with the *p*-value of 5.94e−07 displaying an intense LD with nearby SNPs (Figure 2D).

### Comparison Analyses Between Current and Previous Studies

Among 364 candidate variants, 350 candidate variants were detected by the imputed whole sequence data, while 14 variants were included in BovineHD SNP array (Supplementary Table S2) and 6 of them were reported in other studies. Based on these data, we identified 6 and 11 candidate genes at significant and suggestive threshold levels, respectively (Supplementary Table S2). We summarized the results of bone weight based on high-density SNP array using different analysis methods from many previous studies (Supplementary Table S3) (Xia et al., 2017; Chang et al., 2018; Miao et al., 2018; Chang et al., 2019; An et al., 2020). A total of 39 candidate variants and 22 genomic regions with 35 candidate genes were obtained (Xia et al., 2017; Chang et al., 2018; Miao et al., 2018; Chang et al., 2019; An et al., 2020). Of those, seven genes were detected in the current study, including *LAP3*, *LCORL*, *NCAPG*, *SLIT2*, *FAM13A*, *MED28*, and *DCAF16*. Notably, we newly identified four genes based on the imputed whole sequence data, containing *CPNE8*, *ABCG2*, *TNPO1*, and *PKD2*.

### Gene Set Enrichment Analysis

We performed the enrichment analysis using 39 genes by integrating current and previous studies. These genes were enriched in eight molecular function and seven biological processes, including nucleosome-dependent ATPase activity (GO: 0043167), cellular response to metal ion (GO: 0071248), and nervous system development (GO: 0007399). Several GO terms were related to the calcium transportation, which may be involved in bone development (Supplementary Table S4).

### Fine-Mapping Analyses for Candidate Variants on BTA6 by Bayesian Fine-Mapping Analysis

We found that significant variants for bone weight were located on BTA6 and concentrated in a specific region (37.31–42.19 Mb) with a high LD level. To distinguish whether these variants were the significant candidates due to their causality or because of their high LD with the true candidate, the Bayesian fine-mapping analysis was conducted to estimate the posterior probability of causality and causality *p*-value of each variant in the independent association signals. Four candidate variants including chr6:38460534, chr6:38696766, chr6:40000601, and chr6:41194565 were identified from four independent association signals based on the Bayesian fine-mapping approach, of which the intergenic variant (chr6:38460534) with a *p*-value (causality) of 1.89e-05 and a PPC of 0.6075 was identified as a potential causative variant; meanwhile, *IBSP* and *LAP3* were observed at the 137,231 bp upstream and 114,056 bp downstream of this variant. In addition, we observed another SNP (chr6:38696766) with a *p*-value (causality) of 5.9718e-08 and a PPC of 0.5141 located nearby *MED28*, *DCAF16*, and *NCAPG* (Supplementary Table S5).

## Discussion

The whole genome sequence variants theoretically contain more causative mutations than the SNP array, which make it more robust for the detection of quantitative trait nucleotides (Daetwyler et al., 2014). In the present study, we conducted the whole genome association studies for bone weight using the imputed sequence data, and the Bayesian fine-mapping was performed to precisely map candidate variants (Jiang et al., 2019).

Our results showed that the heritability estimated by the imputed sequencing variants is close to that by the BovineHD SNP array. Consistent with many previous studies, this finding suggested that the density of BovineHD SNP array can fully capture genetic variance, while the sequence variants may provide redundant information due to LD (van Binsbergen et al., 2015; Veerkamp et al., 2016; Zhang C. et al., 2018). Another explanation is that low imputation accuracy may lead to heritability missing estimated by imputed WGS data (Xavier et al., 2016; Song et al., 2019).

Identification of candidate variants for bone weight in beef cattle have been extensively conducted using multiple strategies based on BovineHD SNP array. For instance, using the gene-based association analysis and single marker association study, a previous study revealed 11 potential variants and several important candidate genes affecting bone weight, such as *FAM184B*, *LAP3*, and *NCAPG* (Xia et al., 2017). Another study used the three association models including fixed polygene, random polygene, and composite interval mapping polygene, and identified a total of seven unique variants and five candidate genes associated with bone weight (*C12ORF74*, *LCORL*, *RIMS2*, *WDFY3*, and *FER1L6*) (Chang et al., 2018; Miao et al., 2018; Chang et al., 2019). Moreover, other methods based on LASSO and LMM model including multi-marker association and bin model have also been applied to obtain several important candidate genes for bone weight in previous studies (*RIMS2*, *LCORL*, and *SLIT2*) (An et al., 2020). However, GWAS in previous studies may be biased due to the design of the SNP array; thus, we conducted the GWAS using the imputed whole genome sequencing data and Bayesian fine-mapping to capture more variants for bone weight.

In the present study, we detected a total of 364 candidate variants for bone weight, 350 of them were not included in the BovineHD SNP array. We annotated 11 potential genes including *SLIT2*, *LAP3*, *MED28*, *NCAPG*, *LCORL*, *DCAF16*, *CPNE8*, *FAM13A*, *ABCG2*, *PKD2*, and *TNPO1*. *SLIT2* embedded with two intergenic variants was captured for bone weight using high-density arrays; this gene was reported to be associated with growth and carcass traits including spleen weight (An et al., 2018), birth weight (Smith et al., 2019), bone weight (Xia et al., 2017; An et al., 2020), carcass weight, and eye muscle area (Bhuiyan et al., 2018) in cattle. *LAP3*, *MED28*, *NCAPG*, *LCORL*, and *DCAF16* were identified within the significant genomic region (chr6: 37315342–42194093), captured by 24 candidate variants. *LAP3*, as a member of LAPs family involved in cell maintenance and growth development, had been suggested that it has potential function on body weight in sheep (La et al., 2019) and body measurements traits in cattle (An et al., 2019). Moreover, previous studies also reported that *LAP3* is the potential candidate gene for feed efficiency component traits (Lindholm-Perry et al., 2011; Zhang et al., 2016), carcass merit, and internal organ traits including kidney weight and spleen weight (Song et al., 2016; Xia et al., 2017; An et al., 2018; Miao et al., 2018). *MED28*, a gene involved in the regulation of cell proliferation and cycle (Cho et al., 2019), was captured to associate with body weight, intramuscular fat content, and yearling weight in cattle (Santiago et al., 2017a; Santiago et al., 2017b; Anton et al., 2018). Additionally, two significant intron variants are located within *NCAPG*, which is a well-known gene involved in cell proliferation (Seipold et al., 2009) and has been reported to be associated with feed intake, average daily gain (Lindholm-Perry et al., 2011; Zhang et al., 2016), and carcass traits including hot carcass weight and lean meat yield (Wang et al., 2020). *LCORL* and *DCAF16* were also identified for bone weight in our study, and these two genes have been captured as the candidate genes for calving ease (Bongiorni et al., 2012; Sahana et al., 2015), carcass and meat quality traits, as well as feed efficiency component traits in cattle, which may be directly related to the stature size (Zhang et al., 2016; Anton et al., 2018; Wang et al., 2020).

To evaluate the potential impact of the candidate variants on the gene expression, we queried the TFs existing in the six candidate genes that were located in the BTA6 block based on the AnimalTFDB3.0. Notably, we identified a TF cofactor (Cofactors Family: Mediator complex) related to *MED28* and a TF related to *LCORL* (TF Family: HTH) in *Bos taurus*, which displayed high ortholog identity and conservation across multiple species. Moreover, using the gene expression data obtained from the Bgee database, we observed that *MED28* has a high expression level in prefrontal cortex, brain, etc., but a relatively low expression level in skeletal muscle tissue, and *LCORL* was also observed expressing in testis, colon, brain, and skeletal muscle tissue. In addition, we observed a total of 8,697 regions overlapped with the cattle QTLdb (release 45, August 23, 2021), involved with many economic traits related to the stature size, including average daily gain, body weight, bone weight, and body measurements traits.

Under the suggestive threshold, a total of eight variants were identified nearby *FAM13A* on BTA6; *FAM13A* was captured to be associated with bone weight using a gene-based association method (Xia et al., 2017). Compared with many previous studies, we identified four candidate genes, namely, *CPNE8*, *ABCG2*, *PKD2*, and *TNP O 1*. Of those, *CPNE8* and *ABCG2* were firstly reported for bone weight in this study. *PKD2* was identified by an intron variant (chr6:38024322) with a *p*-value of 4.52e-08, which was related to calcium homeostasis and involved in mitotic cell cycle process (Olsen et al., 2007; Abo-Ismail et al., 2014). *PKD2* was detected as one of the candidate genes for birth weight, weaning weight, and yearling weight in cattle (Weng et al., 2016). Remarkably, *PKD2* was identified nearby the candidate QTLs for bone percentage, meat percentage, and meat-to-bone ratio in previous studies (Gutiérrez-Gil et al., 2009; Abo-Ismail et al., 2014). *TNPO1* was embedded with an intergenic variant (chr20:8728684) with a *p*-value of 5.94e-07; this gene was detected to be associated with birth weight, mature weight, and yearling weight in cattle, which implied its role in regulation of the stature in cattle (Weng et al., 2016).

Using the Bayesian fine-mapping approach, we narrowed the scope of candidate region and identified the putative variants on BTA6. A total of four independent signals were identified in the candidate region, and seven genes nearby the putative variants were captured. Notably, *IBSP* was observed at 137,231 bp upstream of an intergenic variant (chr6:38460534) with posterior probability 0.85; this gene has been reported to relate to the structure of bone matrix in human (Denninger et al., 2015) and body weight in cattle (Santiago et al., 2017a). Moreover, many previous studies suggested that *IBSP* has an impact on the skeletal development in mouse (Rivadeneira et al., 2009), sheep (Matika et al., 2016), and cattle (Gutiérrez-Gil et al., 2012; Xu et al., 2018).

## Conclusion

Using imputed sequencing variants, we identified a total of 350 variants that were not included in BovineHD SNP array in Chinese Simmental beef cattle. Our study revealed several candidate genes and GO terms for bone weight. Moreover, we identified four potential variants on BTA6 by the Bayesian fine-mapping approach. Our findings provide insights into understanding the genetic basis of bone weight that may affect stature size, and these results could be potentially applied in a breeding program in cattle.

## Data Availability

The data underlying this study have been uploaded to Dryad. The raw genotype data are accessible using the following https://doi.org/10.5061/dryad.4qc06.
